# High-dimensional quantile mediation analysis with application to a birth cohort study of mother–newborn pairs

**DOI:** 10.1093/bioinformatics/btae055

**Published:** 2024-01-30

**Authors:** Haixiang Zhang, Xiumei Hong, Yinan Zheng, Lifang Hou, Cheng Zheng, Xiaobin Wang, Lei Liu

**Affiliations:** Center for Applied Mathematics, Tianjin University, Tianjin 300072, China; Department of Population, Family and Reproductive Health, Center On the Early Life Origins of Disease, Johns Hopkins University Bloomberg School of Public Health, Baltimore, MD 21205, United States; Department of Preventive Medicine, Northwestern University, Chicago, IL 60611, United States; Department of Preventive Medicine, Northwestern University, Chicago, IL 60611, United States; Department of Biostatistics, University of Nebraska Medical Center, Omaha, NE 68198, United States; Department of Population, Family and Reproductive Health, Center On the Early Life Origins of Disease, Johns Hopkins University Bloomberg School of Public Health, Baltimore, MD 21205, United States; Department of Pediatrics, Johns Hopkins University School of Medicine, Baltimore, MD 21205, United States; Division of Biostatistics, Washington University in St. Louis, St. Louis, MO 63110, United States

## Abstract

**Motivation:**

There has been substantial recent interest in developing methodology for high-dimensional mediation analysis. Yet, the majority of mediation statistical methods lean heavily on mean regression, which limits their ability to fully capture the complex mediating effects across the outcome distribution. To bridge this gap, we propose a novel approach for selecting and testing mediators throughout the full range of the outcome distribution spectrum.

**Results:**

The proposed high-dimensional quantile mediation model provides a comprehensive insight into how potential mediators impact outcomes via their mediation pathways. This method’s efficacy is demonstrated through extensive simulations. The study presents a real-world data application examining the mediating effects of DNA methylation on the relationship between maternal smoking and offspring birthweight.

**Availability and implementation:**

Our method offers a publicly available and user-friendly function qHIMA(), which can be accessed through the R package HIMA at https://CRAN.R-project.org/package=HIMA.

## 1 Introduction

Mediation analysis is an important statistical technique, elucidating the mechanisms of mediators that bridge the connection between an independent variable (e.g. exposure or treatment) and a dependent variable (e.g. health outcome). With the advancement of data collection technology, high-dimensional data have become increasingly prevalent in various fields. As a result, high-dimensional mediation analysis has piqued the interest of a plethora of researchers recently. For example, Zhang *et al.* (2016) proposed a novel method to estimate and test high-dimensional mediation effects in epigenetic studies. Methods to estimate and test mediation effects with high-dimensional compositional microbiome data have been proposed by researchers like Sohn and Li (2019), Wang *et al.* (2020), Zhang *et al.* (2021a), and Zhang *et al.* (2021b). Djordjilović *et al.* (2019) and Fang *et al.* (2021) studied the group testing for high-dimensional mediation effects. Luo *et al.* (2020) and Zhang *et al.* (2021c) embarked on exploring high-dimensional mediation analysis specifically for the survival outcome. Additionally, Dai *et al.* (2022) and Liu *et al.* (2022) made significant strides in hypothesis testing, with the former devising a multiple-testing procedure for high-dimensional mediation hypotheses and the latter probing into large-scale hypothesis testing for causal mediation effects. Building on previous works, Perera *et al.* (2022) refined estimation and inference procedures for the high-dimensional linear mediation model, extending the foundations laid by Zhang *et al.* (2016). For an expansive understanding of high-dimensional mediation analysis, please refer to two review papers by Zeng *et al.* (2021) and Zhang *et al.* (2022).

It is worth noting that many of the aforementioned studies primarily employed traditional mean-regression methods to analyze outcomes. However, these mean-centric techniques may not fully capture the mediation effects across the entire spectrum of the outcome distribution. For instance, Shen *et al.* (2014) introduced a quantile mediation model focusing on the outcome distribution, with a single mediator modeled through linear regression. Their study found that the mediation effects of walkability on body mass index (BMI) were substantially larger at the upper quantiles as opposed to the median or average. Essentially, individuals with elevated BMI levels might respond differently to interventions compared to those with average BMIs. Relying solely on mean regression could therefore offer an incomplete perspective of mediation effects across various outcome distribution segments. In a parallel vein, Bind *et al.* (2017) delved into the controlled direct and indirect effects of exposure across percentiles of both the mediator and outcome, taking into account longitudinal data.

It is necessary to point out that the models presented by Shen *et al.* (2014) and Bind *et al.* (2017) honed in exclusively on single-mediator cases. Given the prevalence of high-dimensional data, there is a pressing need to develop innovative statistical methods for quantile mediation analysis that can accommodate high-dimensional mediators. The motivation for our work stems from a birth cohort study, examining 954 mother–newborn pairs from a primarily urban, low-income, multi-ethnic US birth cohort. A total of 865 859 CpG sites were measured for each newborn cord blood DNA sample. The study by Xu et al. (2021) revealed that newborns exposed to smoking weighed an average of 258 g less than their unexposed counterparts. Moreover, their findings suggested that the impact of maternal smoking on offspring birthweight is mediated by DNA methylation, as indicated by mean-regression models. Probing into the mediation effects of DNA methylation specifically on low birth weight (LBW) or high birth weight (HBW) individuals, as opposed to those of average weight, introduces a riveting topic of mediation analysis.

As of our current understanding, there are no existing methods in published literature tailored for high-dimensional quantile mediation analysis. In this study, we introduce a novel quantile mediation model that incorporates high-dimensional mediators. Here, the outcome *Y* is characterized by a quantile regression model, while the mediators *M_k_*s are represented through a collection of linear regression models. The advantages of our proposed methodology are manifold. First, individuals experiencing extreme medical outcomes might be at a heightened risk for specific diseases. Traditional regression methods, grounded in averages, might overlook the influences of markers on outcomes at the tails of the distribution. Our quantile-focused mediation models could provide a comprehensive picture of mediating mechanisms that occur at the tails of outcome distributions. Second, we employ a screening procedure to reduce the dimension of mediators, which could considerably reduce the computational burden during the estimation phase. Notably, post-screening survivors predominantly include mediators with significant relevance, elevating the method’s precision. Third, to identify active mediators, we introduce a statistic grounded in a joint significance test, ensuring mediator selection with desired confidence levels.

The remainder of this article is organized as follows: Section 2 unveils our newly developed quantile mediation model, accommodating high-dimensional mediators. We also present a three-step approach for pinpointing the most influential mediators. In Section 3, simulations are carried out to evaluate the performance of our proposed methodology. Section 4 comprises the application of our advanced quantile mediation model to a birth cohort study involving multi-ethnic US mother–newborn pairs. Finally, Section 5 encapsulates our concluding remarks and future directions.

## 2 Model and method

**Figure 1.**
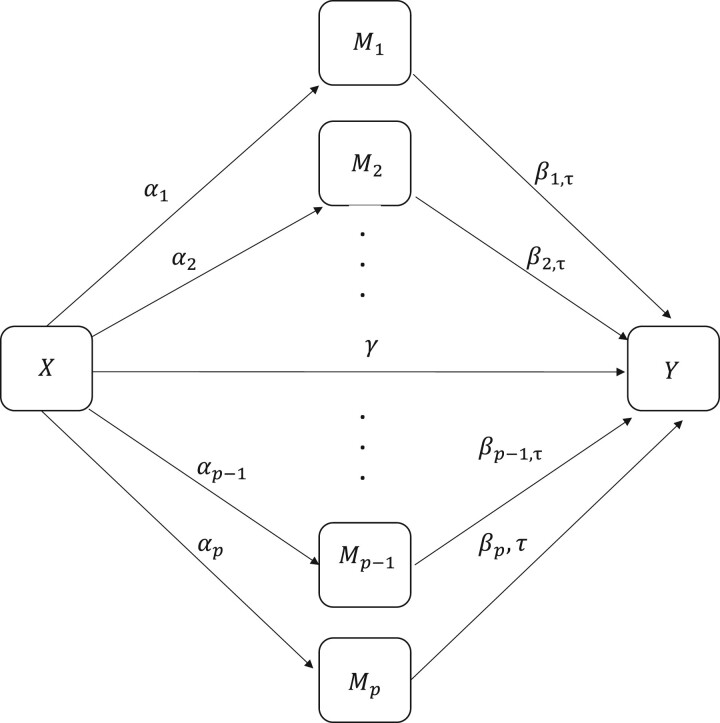
A scenario of high-dimensional quantile mediation model under level τ∈(0,1) (confounding variables omitted).

Let *X*, $M=(M_{1},\ldots ,M_{p})\prime$, $Z=(Z_{1},\ldots ,Z_{q})\prime$, and *Y* represent the observed exposure, mediators, covariates, and outcome, respectively, where *p* is the number of mediators and *q* is the dimension of **Z**. Denote by $Q_{\tau }(Y|Z)$ the *τ*th percentile of the conditional distribution of outcome *Y* given **Z**. Within the potential outcome framework, $M(x)=(M_{1}(x),\ldots ,M_{p}(x))\prime$ represents the potential mediators that would have been observed if *X* had been set to *x*, while Y(x,m) denotes the potential outcome that would have been observed if *X* had been set to *x* and **M** had been set to **m**. Denote by $Q_{\tau }\{Y(x,m)|Z\}$ the *τ*th percentile of the conditional distribution of the potential outcome Y(x,m) given **Z**. In the potential outcome framework, we propose a high-dimensional linear quantile mediation model (Fig. 1), which is presented as follows:

(1) $$M_{k}(x)=c_{k}\;+\;\alpha_{k}x+\zeta_{k}^{\prime }Z+e_{k},\;\;\;k=1,\ldots ,p,$$

(2) $$Q_{\tau }\{Y(x,m)|Z\}=c_{\tau }\;+\;\gamma_{\tau }x\;+\;\beta_{1,\tau }m_{1}\;+\;\cdots \;+\;\beta_{p,\tau }m_{p}\;+\;\eta_{\tau }^{\prime }Z,$$

where $m=(m_{1},\ldots ,m_{p})\prime$ is a high-dimensional vector of mediators; $Z=(Z_{1},\ldots ,Z_{q})\prime$ is a vector of confounding variables or covariates; $\gamma_{\tau }$ is the “direct effect” of *X* on the *τ*th quantile of *Y*, after adjusting for all mediators and covariates; $\alpha =(\alpha_{1},\ldots ,\alpha_{p})\prime$ is a vector of parameters relating the exposure to *p* mediating variables; and $\beta_{\tau }=(\beta_{1,\tau },\ldots ,\beta_{p,\tau })\prime$ is a vector of parameters relating the mediators to the *τ*th quantile of the dependent variable adjusting for the effects of the exposure and covariates; $\zeta_{k}$’s and η are the parameters of covariates. In addition, $c_{\tau }$ and *c_k_*’s are the intercept terms; *e_k_*’s are zero-mean error terms.

Let $Q_{\tau }\{Y(x,E[M(x^{*})|Z])|Z\}$ represent the *τ*th quantile of the conditional distribution of the potential outcome that would have been observed if *X* were set to *x* and **m** were set to its conditional expected counterfactual value $E[M(x^{*})|Z]$. Under the regular assumptions in the Supplementary Materials, we have derived that

$$Q_{\tau }\{Y(x,E[M(x^{*})|Z])|Z\}=C_{1}+\gamma_{\tau }x+\left(\sum_{k=1}^{p}\alpha_{k}\beta_{k,\tau }\right)x^{*}+C_{2}^{\prime }Z,$$

where $C_{1}=c_{\tau }+\sum_{k=1}^{p}c_{k}\beta_{k,\tau }$ and $C_{2}=\eta_{\tau }+\sum_{k=1}^{p}\beta_{k,\tau }\zeta_{k}$. We define the controlled direct effect (CDE) as

$$\begin{array}{c} CDE=Q_{\tau }\{Y(x,E[M(x^{*})|Z])|Z\}-Q_{\tau }\{Y(x^{*},E[M(x^{*})|Z])|Z\}\\ =\gamma_{\tau }(x-x^{*}). \end{array}$$

The controlled indirect effect (CIE) is defined as

(3) $$\begin{array}{c} CIE=Q_{\tau }\{Y(x,E[M(x)|Z])|Z\}-Q_{\tau }\{Y(x,E[M(x^{*})|Z])|Z\}\\ =\sum_{k=1}^{p}\alpha_{k}\beta_{k,\tau }(x-x^{*}). \end{array}$$

Note that the definitions of natural direct effect (NDE) and natural indirect effect (NIE) (in the Supplementary Material) cannot be applied in this context due to our specific focus on outcome quantiles. The CIE through the path $X\to M_{k}\to Q_{\tau }(Y|X,M,Z)$, as expressed by $\alpha_{k}\beta_{k,\tau }$ for k=1,…,p, is derived from (3). This implies that the *τ*th conditional quantile of *Y* serves as the primary outcome of interest. The set $\Omega_{\tau }=\{k:\alpha_{k}\beta_{k,\tau }\neq 0,k=1,\ldots ,p\}$ denotes the indices of statistically significant mediators at a given quantile level τ∈(0,1). Here we assume the sparsity of active mediators with $|\Omega_{\tau }|\ll p$. By virtue of (1) and (2), we can reformulate it as a structural equation model (SEM) to evaluate the mediating effects of high-dimensional mediators on the outcome distribution:

$$\begin{array}{c} M_{k}=c_{k}+\alpha_{k}X+\zeta_{k}^{\prime }Z+e_{k},\;\;\;k=1,\ldots ,p,\\ Q_{\tau }(Y|X,M,Z)=c_{\tau }+\gamma_{\tau }X+\beta_{1,\tau }M_{1}+\cdots +\beta_{p,\tau }M_{p}+\eta_{\tau }^{\prime }Z. \end{array}$$

Assume that the observed samples are *X_i_*, $M_{i}=(M_{i1},\ldots ,M_{ip})\prime$, and *Y_i_*, where i=1,…,n. Our main interest is to provide an estimated index set ${\hat{\Omega }}_{\tau }$ with desirable confidence. To achieve this objective, we propose a three-step approach as outlined below.

**Step 1.** (*Mediator screening*). First, the mediators are standardized to ensure the regression coefficients are on the same scale. For k=1,…,p, we perform a series of marginal quantile models for the *p* mediators:

(4) $$Q_{k,\tau }(Y|X,M_{k},Z)=c_{\tau }+\gamma_{\tau }X+\beta_{k,\tau }M_{k}+\eta_{\tau }^{\prime }Z,$$

Based on the marginal quantile screening idea of Li *et al.* (2023), we can identify a subset $I_{\tau }=\{1\leq k\leq p:$*M_k_* is among the top d=2[n/ log(n)] mediators having the largest $\left|{\tilde{\beta }}_{k}\right|$}, where ${\tilde{\beta }}_{k}$ is the estimate in marginal model (4). The employed marginal quantile screening approach can be considered as a specific parametric case of Li *et al.* (2023), which has demonstrated a high probability that the nonzero $\beta_{k,\tau }$’s belong to $I_{\tau }$. In other words, we can reduce the dimension of mediators from *p* to *d*, while ensuring that active mediators are retained with a probability approaching to one.

**Step 2.** (*Penalized estimate*). Conduct variable selection for mediators ${\left\{M_{k}\right\}}_{k\in I_{\tau }}$ by minimizing the penalty-based criterion,

(5) $$\begin{array}{c} G(\theta_{\tau })=\frac{1}{n}\sum_{i=1}^{n}\rho_{\tau }\left(Y_{i}-c-\gamma_{\tau }X_{i}-\sum_{k\in I_{\tau }}\beta_{k,\tau }M_{ik}-\eta_{\tau }^{\prime }Z_{i}\right)\\ +\sum_{k=1}^{d+q+2}pen(\theta_{k,\tau }), \end{array}$$

where $\theta_{\tau }=(\beta_{I,\tau }^{\prime },c,\gamma_{\tau },\eta_{\tau }^{\prime })\prime \in R^{d+q+2}$ and $\beta_{I,\tau }$ denotes a sub-vector of β with indices belonging to I. $\rho_{\tau }(v)=v\{\tau -I(v<0)\}$ is the check function, and pen(·) is the minimax concave penalty (MCP; Zhang 2010) with the following expression:

$$\begin{array}{c} p_{\lambda ,\delta }(\theta_{k,\tau })=\lambda \left[|\theta_{k,\tau }|-\frac{|\theta_{k,\tau }{|}^{2}}{2\delta \lambda }\right]I\{0\leq |\theta_{k,\tau }|<\delta \lambda \}\\ +\frac{\lambda^{2}\delta }{2}I\{|\theta_{k,\tau }|\geq \delta \lambda \}. \end{array}$$

Here λ>0 is the regularization parameter, and δ>0 determines the concavity of MCP. The MCP procedure for quantile regression has been implemented by Tan *et al.* (2022). In practical applications, the R package conquer can be utilized to solve Equation (5). Let $S_{\tau }=\{k:{\tilde{\beta }}_{k,\tau }\neq 0\}$ denote the index set of survived mediators after Step 2, where ${\tilde{\beta }}_{k,\tau }$’s are the penalized estimates in (5).

**Step 3.** (*Mediator selection*). To conduct mediator selection, we take into account the refitted sub-model as follows:

(6) $$\begin{array}{c} M_{k}=c_{k}+\alpha_{k}X+\zeta_{k}^{\prime }Z+e_{k},\;\;\;k\in S_{\tau },\\ Q_{\tau }(Y|X,M_{S_{\tau }},Z)=c_{\tau }+\gamma_{\tau }X+\sum_{k\in S_{\tau }}\beta_{k,\tau }M_{k}+\eta_{\tau }^{\prime }Z, \end{array}$$

where $M_{S_{\tau }}$ denotes a sub-vector of **M** with indices belonging to $S_{\tau }$. The parameter estimator ${\hat{\beta }}_{k,\tau }$ and its standard error ${\hat{\sigma }}_{\beta_{k}}$ in model (6) can be easily obtained by the R function rq(). The JS-type decision statistic is defined as

(7) $$D_{k,\tau }^{JS}=\min(d_{\tau }P_{k,\tau }^{JS},1),\;\;k\in S_{\tau },$$

where $d_{\tau }$ is the cardinality of set $S_{\tau },\;P_{k,\tau }^{JS}=\max(P_{\alpha_{k}},P_{\beta_{k},\tau }),\;\max(a,b)$ denotes the maximum of *a* and *b*; $P_{\alpha_{k}}=2\{1-\Phi_{N(0,1)}(|{\hat{\alpha }}_{k}|/{\hat{\sigma }}_{\alpha_{k}})\},\;{\hat{\alpha }}_{k}$ is the ordinary least squares estimate with its estimated standard error ${\hat{\sigma }}_{\alpha_{k}}$, and $P_{\beta_{k,\tau }}=2\{1-\Phi_{N(0,1)}(|{\hat{\beta }}_{k,\tau }|/{\hat{\sigma }}_{\beta_{k,\tau }})\}$.

Based on the above three-step approach, an estimated set of indices for significant mediators is derived as ${\hat{\Omega }}_{JS}(\tau )=\{k:D_{k,\tau }^{JS}<0.05,k\in S_{\tau }\}$, where $D_{k,\tau }^{JS}$ is defined in (7). We provide a publicly accessible and user-friendly function qHIMA(), which can be conveniently accessed through the R package HIMA.

## 3 Numerical simulation

In this section, we perform simulations to evaluate the effectiveness of our proposed methodology. For comparison, we also consider the one-mediator methods of Shen *et al.* (2014) and Bind *et al.* (2017) with Bonferroni adjusted *P*-values for multiple comparisons, where we only focus on mediator’s median (τ=0.5) in Bind *et al.* (2017)’s method. The mediators are generated from the following linear models:

(8) $$M_{k}=c_{k}+\alpha_{k}X+\zeta_{k}^{\prime }Z+e_{k},\;\;\;k=1,\ldots ,p,$$

where the exposure *X* follows *N*(0, 4); the covariates $Z=(Z_{1},Z_{2})\prime$ are independently generated from *N*(0, 4); $e=(e_{1},\ldots ,e_{p})\prime$ is generated from a multivariate normal distribution with mean zero and covariance matrix $\Sigma ={\left(0.25^{\left|i-j\right|}\right)}_{i,j}$.

We consider the following two cases for the outcomes:

(9) Case I.   Y=c+γX+β′M+η′Z+ϵ,

(10) Case II.  Y=c+γX+β′M+η′Z+ϵ(θX+ϕ′M),

where *ϵ* follows from *N*(0, 1). Under Case I, the *τ*th conditional quantile of *Y* is given by

$$Q_{\tau }(Y|X,M,Z)=c+Q_{\tau }(\epsilon )+\gamma X+\beta \prime M+\eta \prime Z,$$

where $Q_{\tau }(\epsilon )$ represents the *τ*th quantile of *ϵ*. Under Case II, the *τ*th conditional quantile of *Y* can be expressed as

$$Q_{\tau }(Y|X,M,Z)=c+\{\gamma +\theta Q_{\tau }(\epsilon )\}X+\{\beta +Q_{\tau }(\epsilon )\phi \}\prime M+\eta \prime Z.$$

The true values of α, β and ϕ are set to be α=(0.85,1.2,1,0.15,−0.25,0.65,−0.50,0,…,0)′, β=(0.85,1.2,1,0.25,−0.15,0,0,0.75,−0.5,0,…,0)′ and ϕ=(0.1,0.1,0.1,0,…,0)′, where *p *=* *3000. Moreover, $c_{k}=c=0,\;\gamma =0.5,\;\theta =0.1,\;\zeta_{k}=(0.3,0.3)\prime ,\;\eta =(0.5,0,5)\prime$. The sample size is chosen as n= 200 and 300, respectively. All the simulation results are based on 500 repetitions.

First, we generate random samples with models (8), (9) and (10) to evaluate the proposed method in Section 2. The quantile level is chosen as τ= 0.05, 0.25, 0.5, 0.75, and 0.95, respectively. In Tables 1 and 2, we report the simulation results on the mediation effects ${\left\{\alpha_{k}\beta_{k,\tau }\right\}}_{k=1}^{6}$, including the bias (Bias) given by the difference of sample means of estimators and the true value, together with the sampling standard error (SSE). The estimation results for $\alpha_{k}\beta_{k,\tau }$’s (k=7,…,p) are similar to that of $\alpha_{6}\beta_{6,\tau }$, so we omit the details.

**Table 1.** The (Bias, SSE) of estimation for mediation effects in Case I.

		*n* = 200			*n* = 300		
	$\alpha_{k}\beta_{k,\tau }$	Shen *et al.^a^*	Bind *et al.^b^*	Proposed^c^	Shen *et al.*	Bind *et al.*	Proposed
τ=0.05	$\alpha_{1}\beta_{1,\tau }$	(0.3088, 0.2792)	(0.3065, 0.2793)	(−0.0541, 0.2386)	(0.3233, 0.2275)	(0.3230, 0.2277)	(−0.0173, 0.1266)
	$\alpha_{2}\beta_{2,\tau }$	(0.5335, 0.3489)	(0.5317, 0.3515)	(−0.0479, 0.2593)	(0.5598, 0.2632)	(0.5576, 0.2659)	(−0.0406, 0.1885)
	$\alpha_{3}\beta_{3,\tau }$	(0.4132, 0.3126)	(0.4168, 0.3165)	(−0.0059, 0.2308)	(0.4249, 0.2711)	(0.4259, 0.2749)	(0.0201, 0.1605)
	$\alpha_{4}\beta_{4,\tau }$	(0.0453, 0.0608)	(0.0444, 0.0628)	(−0.0305, 0.0213)	(0.0487, 0.0437)	(0.0484, 0.0446)	(−0.0258, 0.0235)
	$\alpha_{5}\beta_{5,\tau }$	(−0.0411, 0.0975)	(−0.0418, 0.0991)	(−0.0377, 0.0055)	(−0.0438, 0.0772)	(−0.0436, 0.0774)	(−0.0374, 0.0017)
	$\alpha_{6}\beta_{6,\tau }$	(0.0298, 0.2551)	(0.0303, 0.2559)	(0.0009, 0.0175)	(0.0257, 0.2024)	(0.0253, 0.2021)	(0.0013, 0.0169)
τ=0.25	$\alpha_{1}\beta_{1,\tau }$	(0.3084, 0.1877)	(0.3064, 0.1906)	(−0.0172, 0.1123)	(0.3154, 0.1459)	(0.3152, 0.1474)	(−0.0120, 0.0866)
	$\alpha_{2}\beta_{2,\tau }$	(0.5641, 0.2174)	(0.5624, 0.2232)	(−0.0465, 0.1657)	(0.5749, 0.1806)	(0.5727, 0.1846)	(−0.0204, 0.1326)
	$\alpha_{3}\beta_{3,\tau }$	(0.3909, 0.2063)	(0.3944, 0.2097)	(0.0135, 0.1390)	(0.4143, 0.1604)	(0.4148, 0.1614)	(0.0085, 0.1186)
	$\alpha_{4}\beta_{4,\tau }$	(0.0439, 0.0411)	(0.0429, 0.0449)	(−0.0281, 0.0202)	(0.0481, 0.0355)	(0.0482, 0.0372)	(−0.0188, 0.0224)
	$\alpha_{5}\beta_{5,\tau }$	(−0.0349, 0.0597)	(−0.0349, 0.0602)	(−0.0375, 0.0001)	(−0.0424, 0.0508)	(−0.0425, 0.0507)	(−0.0375, 0.0006)
	$\alpha_{6}\beta_{6,\tau }$	(0.0256, 0.1496)	(0.0255, 0.1489)	(0.0007, 0.0083)	(0.0225, 0.1260)	(0.0225, 0.1259)	($<10^{-4}$, 0.0073)
τ=0.5	$\alpha_{1}\beta_{1,\tau }$	(0.3221, 0.1734)	(0.3210, 0.1761)	(−0.0195, 0.1065)	(0.3110, 0.1316)	(0.3109, 0.1321)	(−0.0171, 0.0881)
	$\alpha_{2}\beta_{2,\tau }$	(0.5728, 0.2048)	(0.5719, 0.2092)	(−0.0577, 0.1697)	(0.5736, 0.1737)	(0.5706, 0.1782)	(−0.0409, 0.1312)
	$\alpha_{3}\beta_{3,\tau }$	(0.4069, 0.1949)	(0.4107, 0.1976)	(−0.0035, 0.1395)	(0.4077, 0.1448)	(0.4080, 0.1488)	−0.0063, 0.1114)
	$\alpha_{4}\beta_{4,\tau }$	(0.0455, 0.0391)	(0.0452, 0.0435)	(−0.0250, 0.0222)	(0.0476, 0.0344)	(0.0476, 0.0363)	(−0.0158, 0.0216)
	$\alpha_{5}\beta_{5,\tau }$	(−0.0374, 0.0565)	(−0.0373, 0.0564)	(−0.0375, $<10^{-4}$)	(−0.0380, 0.0441)	(−0.0379, 0.0439)	(−0.0374, 0.0018)
	$\alpha_{6}\beta_{6,\tau }$	(0.0215, 0.1444)	(0.0212, 0.1440)	($<10^{-4},<10^{-4}$)	(0.0265, 0.1143)	(0.0267, 0.1142)	(0.0006, 0.0067)
τ=0.75	$\alpha_{1}\beta_{1,\tau }$	(0.3266, 0.1878)	(0.3246, 0.1904)	(−0.0234, 0.1202)	(0.3108, 0.1517)	(0.3110, 0.1545)	(−0.0079, 0.0873)
	$\alpha_{2}\beta_{2,\tau }$	(0.5704, 0.2246)	(0.5684, 0.2291)	(−0.0526, 0.1839)	(0.5609, 0.1870)	(0.5577, 0.1904)	(−0.0343, 0.1462)
	$\alpha_{3}\beta_{3,\tau }$	(0.4168, 0.2021)	(0.4202, 0.2048)	(0.0051, 0.1452)	(0.4030, 0.1659)	(0.4030, 0.1672)	(0.0069, 0.1134)
	$\alpha_{4}\beta_{4,\tau }$	(0.0482, 0.0428)	(0.0463, 0.0447)	(−0.0271, 0.0214)	(0.0464, 0.0353)	(0.0463, 0.0373)	(−0.0190, 0.0225)
	$\alpha_{5}\beta_{5,\tau }$	(−0.0371, 0.0599)	(−0.0369, 0.0603)	(−0.0374, 0.0012)	(−0.0388, 0.0498)	(−0.0387, 0.0498)	(−0.0374, 0.0019)
	$\alpha_{6}\beta_{6,\tau }$	(0.0155, 0.1629)	(0.0156, 0.1626)	(0.0004, 0.0104)	(0.0287, 0.1251)	(0.0289, 0.1253)	(0.0009, 0.0099)
τ=0.95	$\alpha_{1}\beta_{1,\tau }$	(0.3204, 0.2905)	(0.3185, 0.2923)	(−0.0693, 0.2358)	(0.2965, 0.2315)	(0.2969, 0.2353)	(−0.0135, 0.1482)
	$\alpha_{2}\beta_{2,\tau }$	(0.5710, 0.3364)	(0.5693, 0.3405)	(−0.0338, 0.2565)	(0.5766, 0.2612)	(0.5733, 0.2631)	(−0.0397, 0.1941)
	$\alpha_{3}\beta_{3,\tau }$	(0.4032, 0.3170)	(0.4065, 0.3195)	(−0.0171, 0.2309)	(0.3939, 0.2703)	(0.3941, 0.2721)	(0.0115, 0.1644)
	$\alpha_{4}\beta_{4,\tau }$	(0.0489, 0.0593)	(0.0475, 0.0623)	(−0.0315, 0.0193)	(0.0479, 0.0481)	(0.0478, 0.0500)	(−0.0273, 0.0213)
	$\alpha_{5}\beta_{5,\tau }$	(−0.0376, 0.0957)	(−0.0381, 0.0953)	(−0.0374, 0.0033)	(−0.0442, 0.0788)	(−0.0439, 0.0799)	(−0.0375, 0.0010)
	$\alpha_{6}\beta_{6,\tau }$	(0.0267, 0.2408)	(0.0267, 0.2405)	(0.0007, 0.0135)	(0.0240, 0.2015)	(0.0243, 0.2025)	(0.0008, 0.0139)

a “Shen et al” denotes the method of Shen *et al.* (2014); ^b^ “Bind *et al.*” denotes the method of Bind *et al.* (2017); ^c^ “Proposed” denotes our method.

**Table 2.** The (Bias, SSE) of estimation for mediation effects in Case II.

		*n* = 200			*n* = 300		
	$\alpha_{k}\beta_{k,\tau }$	Shen *et al.^a^*	Bind *et al.^b^*	Proposed^c^	Shen *et al.*	Bind *et al.*	Proposed
τ=0.05	$\alpha_{1}\beta_{1,\tau }$	(0.4661, 0.2804)	(0.4654, 0.2843)	(0.0885, 0.2472)	(0.4632, 0.2286)	(0.4629, 0.2287)	(0.1178, 0.1519)
	$\alpha_{2}\beta_{2,\tau }$	(0.7665, 0.3709)	(0.7653, 0.3724)	(0.1642, 0.2655)	(0.7732, 0.2668)	(0.7699, 0.2679)	(0.1546, 0.2065)
	$\alpha_{3}\beta_{3,\tau }$	(0.5795, 0.3336)	(0.5839, 0.3395)	(0.1625, 0.2309)	(0.5777, 0.2591)	(0.5781, 0.2625)	(0.1689, 0.1774)
	$\alpha_{4}\beta_{4,\tau }$	(0.0498, 0.0607)	(0.0498, 0.0649)	(−0.0336, 0.0162)	(0.0469, 0.0481)	(0.0467, 0.0501)	(−0.0257, 0.0229)
	$\alpha_{5}\beta_{5,\tau }$	(−0.0383, 0.0998)	(−0.0384, 0.1021)	(−0.0375, $<10^{-4}$)	(−0.0427, 0.0779)	(−0.0425, 0.0782)	(−0.0375, $<10^{-4}$)
	$\alpha_{6}\beta_{6,\tau }$	(0.0421, 0.2479)	(0.0426, 0.2487)	(0.0022, 0.0265)	(0.0261, 0.1907)	(0.0259, 0.1911)	(−0.0001, 0.0158)
τ=0.25	$\alpha_{1}\beta_{1,\tau }$	(0.3692, 0.1844)	(0.3683, 0.1877)	(0.0506, 0.0868)	(0.3685, 0.1392)	(0.3684, 0.1411)	(0.0532, 0.0694)
	$\alpha_{2}\beta_{2,\tau }$	(0.6356, 0.2143)	(0.6348, 0.2203)	(0.0416, 0.1383)	(0.6473, 0.1732)	(0.6442, 0.1779)	(0.0580, 0.1083)
	$\alpha_{3}\beta_{3,\tau }$	(0.4656, 0.2055)	(0.4695, 0.2091)	(0.0867, 0.1151)	(0.4709, 0.1567)	(0.4713, 0.1609)	(0.0739, 0.0860)
	$\alpha_{4}\beta_{4,\tau }$	(0.0453, 0.0367)	(0.0449, 0.0412)	(−0.0259, 0.0194)	(0.0461, 0.0331)	(0.0459, 0.0349)	(−0.0159, 0.0198)
	$\alpha_{5}\beta_{5,\tau }$	(−0.0366, 0.0563)	(−0.0363, 0.0569)	(−0.0375, $<10^{-4}$)	(−0.0388, 0.0495)	(−0.0388, 0.0492)	(−0.0375, $<10^{-4}$)
	$\alpha_{6}\beta_{6,\tau }$	(0.0291, 0.1557)	(0.0294, 0.1552)	(0.0007, 0.0119)	(0.0270, 0.1243)	(0.0269, 0.1241)	($<10^{-4}$, 0.0018)
τ=0.5	$\alpha_{1}\beta_{1,\tau }$	(0.3134, 0.1662)	(0.3124, 0.1691)	(−0.0058, 0.0765)	(0.3050, 0.1315)	(0.3050, 0.1337)	(−0.0081, 0.0563)
	$\alpha_{2}\beta_{2,\tau }$	(0.5602, 0.1948)	(0.5593, 0.2003)	(−0.0370, 0.1189)	(0.5599, 0.1545)	(0.5569, 0.1595)	(−0.0294, 0.0867)
	$\alpha_{3}\beta_{3,\tau }$	(0.3981, 0.1865)	(0.4021, 0.1904)	(0.0118, 0.1038)	(0.4034, 0.1389)	(0.4038, 0.1441)	(−0.0031, 0.0685)
	$\alpha_{4}\beta_{4,\tau }$	(0.0453, 0.0376)	(0.0450, 0.0429)	(−0.0217, 0.0211)	(0.0453, 0.0319)	(0.0452, 0.0341)	(−0.0105, 0.0166)
	$\alpha_{5}\beta_{5,\tau }$	(−0.0373, 0.0545)	(−0.0371, 0.0551)	(−0.0375, $<10^{-4}$)	(−0.0384, 0.0431)	(−0.0384, 0.0430)	(−0.0374, 0.0021)
	$\alpha_{6}\beta_{6,\tau }$	(0.0223, 0.1457)	(0.0223, 0.1456)	(−0.0001, 0.0019)	(0.0279, 0.1174)	(0.0281, 0.1173)	(0.0003, 0.0047)
τ=0.75	$\alpha_{1}\beta_{1,\tau }$	(0.2671, 0.1817)	(0.2650, 0.1836)	(−0.0660, 0.0862)	(0.2489, 0.1459)	(0.2490, 0.1480)	(−0.0627, 0.0718)
	$\alpha_{2}\beta_{2,\tau }$	(0.4726, 0.2218)	(0.4708, 0.2269)	(−0.1138, 0.1370)	(0.4747, 0.1657)	(0.4718, 0.1722)	(−0.1147, 0.1044)
	$\alpha_{3}\beta_{3,\tau }$	(0.3474, 0.1993)	(0.3508, 0.2018)	(−0.049, 0.1157)	(0.3314, 0.1591)	(0.3317, 0.1629)	(−0.0617, 0.0887)
	$\alpha_{4}\beta_{4,\tau }$	(0.0483, 0.0406)	(0.0469, 0.0444)	(−0.0247, 0.0214)	(0.0466, 0.0338)	(0.0463, 0.0352)	(−0.0150, 0.0200)
	$\alpha_{5}\beta_{5,\tau }$	(−0.0396, 0.0583)	(−0.0394, 0.0587)	(−0.0375, $<10^{-4}$)	(−0.0419, 0.0503)	(−0.0418, 0.0500)	(−0.0375, $<10^{-4}$)
	$\alpha_{6}\beta_{6,\tau }$	(0.0213, 0.1506)	(0.0215, 0.1503)	(0.0011, 0.0119)	(0.0211, 0.1209)	(0.0214, 0.1211)	(0.0003, 0.0048)
τ=0.95	$\alpha_{1}\beta_{1,\tau }$	(0.1792, 0.3022)	(0.1781, 0.3037)	(−0.2003, 0.2432)	(0.1741, 0.2423)	(0.1744, 0.2448)	(−0.1597, 0.1563)
	$\alpha_{2}\beta_{2,\tau }$	(0.3404, 0.3392)	(0.3395, 0.3423)	(−0.2469, 0.2552)	(0.3661, 0.2686)	(0.3640, 0.2715)	(−0.2496, 0.2079)
	$\alpha_{3}\beta_{3,\tau }$	(0.2294, 0.3216)	(0.2333, 0.3244)	(−0.1694, 0.2431)	(0.2439, 0.2589)	(0.2441, 0.2574)	(−0.1613, 0.1722)
	$\alpha_{4}\beta_{4,\tau }$	(0.0488, 0.0584)	(0.0477, 0.0627)	(−0.0314, 0.0211)	(0.0493, 0.0515)	(0.0492, 0.0528)	(−0.0256, 0.0234)
	$\alpha_{5}\beta_{5,\tau }$	(−0.0463, 0.0944)	(−0.0463, 0.0955)	(−0.0374, 0.0023)	(−0.0467, 0.0771)	(−0.0465, 0.0777)	(−0.0373, 0.0039)
	$\alpha_{6}\beta_{6,\tau }$	(0.0463, 0.2389)	(0.0459, 0.2382)	(0.0008, 0.0165)	(0.0239, 0.1942)	(0.0243, 0.1942)	(0.0005, 0.0103)

a “Shen *et al.*” denotes the method of Shen *et al.* (2014); ^b^ “Bind *et al.*” denotes the method of Bind *et al.* (2017); ^c^ “Proposed” denotes our method.

The results in Tables 1 and 2 demonstrate that our method exhibits significantly smaller Bias and SSE compared to Shen *et al.* and Bind *et al.* Hence, the one-by-one mediator procedure is not applicable for estimating high-dimensional quantile mediation effects. To describe the accuracy of our mediator selection method, we report the following three results:

Model size (MS): $\left|{\hat{\Omega }}_{0}(\tau )\right|$, where $\left|{\hat{\Omega }}_{0}(\tau )\right|$ denotes the number of elements in ${\hat{\Omega }}_{0}(\tau )$;

True positive proportion (TPP): $\left|{\hat{\Omega }}_{0}(\tau )\cap \Omega_{0}(\tau )|/|\Omega_{0}(\tau )\right|$;

False discovery proportion (FDP): $\left|{\hat{\Omega }}_{0}(\tau )\setminus \Omega_{0}(\tau )|/|{\hat{\Omega }}_{0}(\tau )\right|$, where $\left|{\hat{\Omega }}_{0}(\tau )\setminus \Omega_{0}(\tau )\right|$ denotes the set difference of ${\hat{\Omega }}_{0}(\tau )$ and $\Omega_{0}(\tau )$.

Tables 3 and 4 present the mediator selection results in terms of MS, TPP, and FDP. These results suggest that Shen *et al.* and Bind *et al.* tend to select a smaller model with lower TPP and FDP, i.e. the one-by-one mediator procedure is more conservative than our JS-based procedure. Moreover, all methods become better as the sample size *n* increases. Overall, the proposed mediator selection method works well for high-dimensional quantile mediation model in practical applications. From the view of computational efficiency, our method takes approximately 245.34 seconds for one repetition with Case I and *n *=* *300, where the computation is implemented on a laptop with 8G memory.

**Table 3.** The performance of mediator selection in Case I.

		*n* = 200			*n* = 300		
Method	Quantile level	MS	TPP	FDP	MS	TPP	FDP
Shen *et al.*	τ=0.05	1.8520	0.3696	0.0017	2.4700	0.4932	0.0012
	τ=0.25	2.8380	0.5676	$<10^{-4}$	3.0980	0.6196	$<10^{-4}$
	τ=0.50	2.9460	0.5892	$<10^{-4}$	3.1600	0.6316	0.0005
	τ=0.75	2.9160	0.5832	$<10^{-4}$	3.1000	0.6188	0.0017
	τ=0.95	1.8480	0.3696	$<10^{-4}$	2.4460	0.4876	0.0021
Bind *et al.*	τ=0.05	1.8380	0.3672	0.0010	2.4600	0.4912	0.0012
	τ=0.25	2.8220	0.5644	$<10^{-4}$	3.0580	0.6116	$<10^{-4}$
	τ=0.50	2.9220	0.5840	0.0005	3.0760	0.6148	0.0005
	τ=0.75	2.8820	0.5764	$<10^{-4}$	3.0460	0.6080	0.0017
	τ=0.95	1.8480	0.3692	0.0005	2.4340	0.4844	0.0031
Proposed	τ=0.05	2.7080	0.5408	0.0010	3.0320	0.6040	0.0030
	τ=0.25	3.0440	0.6056	0.0040	3.2620	0.6488	0.0042
	τ=0.50	3.0540	0.6084	0.0030	3.3220	0.6612	0.0036
	τ=0.75	3.0100	0.6012	0.0010	3.2500	0.6460	0.0046
	τ=0.95	2.7240	0.5432	0.0022	3.0000	0.5964	0.0045

a “MS” denotes the model size; “TPP” denotes true positive proportion; “FDP” denotes the false discovery proportion; “Shen *et al.*” denotes the method of Shen *et al.* (2014); “Bind *et al.*” denotes the method of Bind *et al.* (2017); “Proposed” denotes our method.

**Table 4.** The performance of mediator selection in Case II.

		*n* = 200			*n* = 300		
Method	Quantile level	MS	TPP	FDP	MS	TPP	FDP
Shen *et al.*	τ=0.05	1.8720	0.3744	$<10^{-4}$	2.4720	0.4944	$<10^{-4}$
	τ=0.25	2.8520	0.5700	0.0005	3.1340	0.6256	0.0013
	τ=0.50	2.9880	0.5972	0.0004	3.1640	0.6320	0.0010
	τ=0.75	2.9360	0.5868	0.0005	3.1260	0.6252	$<10^{-4}$
	τ=0.95	1.8420	0.3684	$<10^{-4}$	2.4720	0.4944	$<10^{-4}$
Bind *et al.*	τ=0.05	1.8660	0.3732	$<10^{-4}$	2.4460	0.4892	$<10^{-4}$
	τ=0.25	2.8340	0.5664	0.0005	3.0640	0.6116	0.0014
	τ=0.50	2.9580	0.5912	0.0005	3.0800	0.6152	0.0010
	τ=0.75	2.9060	0.5808	0.0005	3.0600	0.6120	$<10^{-4}$
	τ=0.95	1.8300	0.3660	$<10^{-4}$	2.4480	0.4892	0.0007
Proposed	τ=0.05	2.7340	0.5452	0.0022	3.0140	0.6008	0.0025
	τ=0.25	3.1200	0.6224	0.0020	3.4140	0.6800	0.0031
	τ=0.50	3.2480	0.6476	0.0024	3.6680	0.7308	0.0031
	τ=0.75	3.1600	0.6304	0.0020	3.4600	0.6888	0.0039
	τ=0.95	2.7260	0.5448	0.0005	3.0320	0.6028	0.0045

a “MS” denotes the model size; “TPP” denotes true positive proportion; “FDP” denotes the false discovery proportion; “Shen *et al.*” denotes the method of Shen *et al.* (2014); “Bind *et al.*” denotes the method of Bind *et al.* (2017); “Proposed” denotes our method.

## 4 A birth cohort study of mother–newborn pairs

Each year in the USA, maternal smoking affects over half a million pregnancies, resulting in fetal growth limitations. This is evident from the reduced birthweight and its subsequent long-term implications as noted by Wang *et al.* (2002). In this section, we apply our proposed methodology to a birth cohort study comprising mother–newborn pairs from a predominantly urban, low-income multi-ethnic birth cohort in the USA.

The study comprised a total of 954 mother–newborn pairs from the racially diverse Boston Birth Cohort (Pearson *et al.* 2022). Among the mothers, 165 (17.3%) had a history of smoking either before or during pregnancy. Newborns exposed to smoking exhibited an average birthweight reduction of 258 g compared to those without exposure (Xu *et al.* 2021). The DNA methylation (DNAm) markers of each newborn were measured using the Illumina Infinium MethylationEPIC BeadChip, which successfully produced DNAm profiles for 865 859 CpG sites (*P *=* *865 859). For each CpG site examined, a *β*-value ranging from 0 to 1 was reported. We aim to investigate the mediating role of DNA methylation (DNAm) markers in the association between smoking (binary exposure: 1 = smoker and 0 = non-smoker) and birthweight (outcome). We also adjust for confounding variables, including maternal age at delivery; parity (not including the index pregnancy): 0 versus 1 or more; maternal education: high school or less versus some college or more; maternal self-reported race: Black versus non-Black, where Black included self-reported Black (African American and Haitian) and non-Black included white and Hispanic; maternal alcohol consumption during pregnancy: never versus ever; maternal pre-pregnancy BMI; child’s sex: female versus male; and gestational age at birth. We also adjust for estimated cord blood cell composition, which was calculated using the estimateCellCounts() function in the “minf” package, with seven cell types: CD4+, T cells, B cells, monocytes, granulocytes, natural killer cells, and nucleated red blood cells.

**Figure 2.**
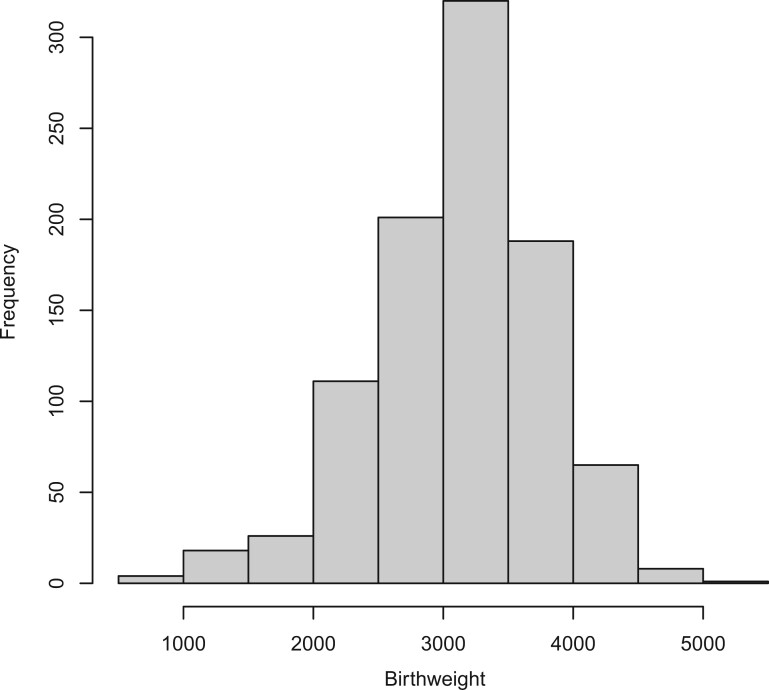
The histogram of infant birth weight (in grams).

We illustrate the distribution of infant birth weight in Fig. 2. We are interested in examining the mediation effects across the entire spectrum of the outcome distribution. In other words, we are looking beyond just the average outcome distribution, as is typically done using mean-regression methods. Specifically, we are keen on the effects on both the low birth weight (LBW) and high birth weight (HBW) samples. Based on the distribution, we can roughly categorize τ∈{0.2,0.3} as the LBW group, τ∈{0.4,0.5,0.6} as the normal weight group, and τ∈{0.7,0.8} as the HBW group. The results from our method for the chosen mediators are summarized in Table 5. In contrast, with the same dataset, the methods by Shen *et al.* (2014) and Bind *et al.* (2017) cannot select any significant mediators after adjusting for multiple comparisons. Under τ∈{0.2,0.3} (i.e. LBW group), we identified three CpG sites (cg25325512, cg07814318, and cg14541773) as significant mediators, while under τ∈{0.4,0.5,0.6} (i.e. regular weight group), one CpG site (cg07814318) is consistently identified to be significant mediators. Studies have shown that the effect of maternal smoking on birth weight is partly mediated by the methylation of cg25325512 (*PIM1*), which contributes to a decrease in birth weight. Several studies have also identified that maternal smoking may influence DNA methylation at the *KLF13* gene region in offsprings. This association may be dose dependent, with stronger effects observed with higher smoking intensity and duration. Additionally, the studies indicate that the association between *KLF13* methylation and smoking may have implications for child health and the developmental origins of the metabolic syndrome. Under τ∈{0.7,0.8} (i.e. HBW group), no significant mediator is identified under τ=0.7, but under τ=0.8, our method identifies CpG sites cg04411342 and cg05575921 as significant mediators. cg05575921 (within gene *AHRR*) exhibits a significant mediating effect in the higher quantiles (HBW), while no mediating effect is observed in the lower quantiles (LBW) and median. Our previous study has identified CpG site cg05575921 as a significant mediator of maternal smoking and birthweight (Xu *et al.* 2021). A low level of methylation at the cg05575921 locus in the *AHRR* gene has been robustly associated with smoking (Grieshober *et al.* 2020). This hypomethylation is thought to mediate the association between maternal smoking and metabolic profiles in children. Functionally, cigarette smoke contains toxic components, such as polycyclic aromatic hydrocarbons, which can induce aryl hydrocarbon receptor (AHR)-mediated *AHRR* expression and methylation (Tantoh *et al.* 2020). These results demonstrate the capability of our method to thoroughly capture the complex mediating effects across different sections of the outcome distribution.

**Table 5.** Summary results of selected CpGs in the birth cohort study.

Quantile ($Q_{\tau }$)	CpG	Position	Gene	${\hat{\alpha }}_{k}$ (SE)	${\hat{\beta }}_{k,\tau }$ (SE)	${\hat{\alpha }}_{k}{\hat{\beta }}_{k,\tau }$
τ=0.2 (2642.0)	cg25325512	chr6	*PIM1*	−0.26702 (0.08792)	0.07428 (0.02167)	−0.01983
	cg07814318	chr15	*KLF13*	−0.24828 (0.06781)	0.10808 (0.03080)	−0.02683
τ=0.3 (2855.0)	cg14541773	chr1	–	−0.39719 (0.08463)	0.11209 (0.03349)	−0.04452
τ=0.4 (3035.0)	cg07814318	chr15	*KLF13*	−0.24828 (0.06781)	0.10653 (0.03249)	−0.02645
τ=0.5 (3177.5)	cg07814318	chr15	*KLF13*	−0.24828 (0.06781)	0.11949 (0.03614)	−0.02967
τ=0.6 (3308.0)	cg07814318	chr15	*KLF13*	−0.24828 (0.06781)	0.15615 (0.03351)	−0.03877
τ=0.8 (3674.0)	cg04411342	chr3	*TMEM108*	−0.28863 (0.08484)	0.10598 (0.03178)	−0.03059
	cg05575921	chr5	*AHRR*	−0.74844 (0.08166)	0.08918 (0.02454)	−0.06675

a τ∈{0.2,0.3} is referred to as the low birth weight group; τ∈{0.8} is the high birth weight group; $Q_{\tau }$ denotes the *τ*th quantile of birth weight data (in grams).

Per the suggestion of a reviewer, in Supplementary Table SA, we present a summary of the top five CpGs identified by Shen *et al.* and Bind *et al.* in their application to the real data. When we compare these results with those obtained through our method in Table 5, numerous discrepancies are evident. For instance, many of the CpGs identified by our method, such as cg25325512, cg07814318, cg04411342, do not appear in the top five lists of Shen *et al.* and Bind *et al.* However, there are some points of agreement. At τ=0.3, cg14541773 is selected both by our method and by Shen *et al.*; cg05575921 is included at τ∈{0.4,0.5} by Shen *et al.* and Bind *et al.*, while our method identifies cg05575921 at τ=0.8.

## 5 Concluding remarks

This study introduced a novel high-dimensional quantile mediation model. A three-step procedure was employed to identify active mediators. Simulations and real data application were conducted to demonstrate the practical utility of our approach. The R function qHIMA() was developed to facilitate the implementation of our method in practice, providing a user-friendly interface. Assumption (C.4) highlights that while mediators may be correlated, they do not have causal relationships with each other. This is a crucial assumption for the validity of the causal interpretations of our Conditional Direct Effect (CDE) and Conditional Indirect Effect (CIE), even though exploring the causal links between the mediators themselves is not our focus.

There are several topics that will be studied in the future. First, the present study sheds light on high-dimensional quantile mediation analysis, which has the potential for further expansion to include survival outcomes (Zhang *et al.* 2021c) and longitudinal data (Bind *et al.* 2017). Second, the proposed method incorporates linear models for the mediators and a quantile model for the outcome. It is desirable to consider using quantile models for both mediators and outcomes under our framework. Third, the development of a robust high-dimensional mediation analysis procedure becomes intriguing when potential outliers are present in the mediators and outcomes. Fourth, the investigation of interactions between exposure and high-dimensional mediators poses significant challenges, necessitating further endeavors to address this issue. Fifth, we have imposed a sparsity condition for active mediators when applying our method. However, the approach to handling quantile mediation effects remains uncertain in situations where there are multiple true mediators that exceed the sample size.

## Supplementary Material

btae055_Supplementary_Data

## Data Availability

The data underlying this article cannot be shared publicly due to ongoing follow-up and the informed consent governing the protection of the privacy of individuals that participated in the study. The data will be shared on reasonable request to the corresponding author.
