# Supplementary material for: High-dimensional quantile mediation analysis with application to a birth cohort study of mother–newborn pairs
Source: Bioinformatics. 2024 Jan 30;40(2):btae055. doi: 10.1093/bioinformatics/btae055 (PMC10873903; doi:10.1093/bioinformatics/btae055)
Supplement: btae055_Supplementary_Data [file btae055_supplementary_data.pdf]

Supplementary Materials for

High-Dimensional Quantile Mediation Analysis with Application to a Birth Cohort Study of Mother–Newborn Pairs

Haixiang Zhang, Xiumei Hong, Yinan Zheng, Lifang Hou,
Cheng Zheng, Xiaobin Wang, and Lei Liu

1 Assumptions, NDE and NIE

For the purpose of identifying causal effects, the causal mediation analysis requires four fundamental assumptions as follows:

(C.1) Stable Unit Treatment Value Assumption (SUTVA). There are no multiple versions of exposures and no interference between subjects, which implies that the observed variables are identical to the potential variables corresponding to the actually observed exposure level. i.e., $\mathbf{M} = \sum_x \mathbf{M}(x) I(X = x)$, and $Y = \sum_x \sum_{\mathbf{m}} Y(x, \mathbf{m}) I(X = x, \mathbf{M} = \mathbf{m})$, where $I(\cdot)$ is the indicator function.

(C.2) There are no measurement errors in the mediators and the outcome.

(C.3) (i) $Y(x, \mathbf{m}) \perp X | \mathbf{Z}$, i.e., no unmeasured confounding between exposure and the potential outcome. (ii) $Y(x, \mathbf{m}) \perp \mathbf{M} | \{X, \mathbf{Z}\}$, i.e., no unmeasured confounding for the mediator-outcome relationship after adjusting for the exposure. (iii) $\mathbf{M}(x) \perp X | \mathbf{Z}$, i.e., no unmeasured confounding for the exposure effect on all the mediators. (iv) $Y(x, \mathbf{m}) \perp \mathbf{M}(x^*) | \mathbf{Z}$, i.e., no exposure-induced confounding between mediators and the potential outcome.

(C.4) The mediators are assumed to be causally independent. i.e., it is not allowed that one mediator is the cause of another.

For convenience, we first review some fundamental concepts of NDE and NIE within the framework of causal mediation analysis.

- Natural direct effect (NDE):

$$\begin{aligned}\text{NDE} &= E[Y(x,\mathbf{M}(x^*))] - E[Y(x^*,\mathbf{M}(x^*))] \\ &= \int_{\mathbf{m}} \{E(Y|x,\mathbf{m}) - E(Y|x^*,\mathbf{m}\}P(\mathbf{M}=\mathbf{m}|x^*)d\mathbf{m}.\end{aligned}$$

- Natural indirect effect (NIE):

$$\begin{aligned}\text{NIE} &= E[Y(x,\mathbf{M}(x))] - E[Y(x,\mathbf{M}(x^*))] \\ &= \int_{\mathbf{m}} E(Y|x,\mathbf{m})\{P(\mathbf{M}=\mathbf{m}|x) - P(\mathbf{M}=\mathbf{m}|x^*)\}d\mathbf{m}.\end{aligned}$$

2 Derivation of $Q_\tau\{Y(x,E[\mathbf{M}(x^*)|\mathbf{Z}])|\mathbf{Z}\}$

Under the assumptions (C.1)-(C.4), the τth percentile of the potential outcome Y distribution setting $X=x$ and $\mathbf{M}=E[\mathbf{M}(x^*)|\mathbf{Z}]$ conditional on the covariate $\mathbf{Z}$ is

$$\begin{aligned}Q_\tau\{Y(x,E[\mathbf{M}(x^*)|\mathbf{Z}])|\mathbf{Z}\} &= Q_\tau\{Y(x,c_1+\alpha_1 x^* + \boldsymbol{\zeta}_1'\mathbf{Z},\cdots,c_p+\alpha_p x^*+\boldsymbol{\zeta}_p'\mathbf{Z})|\mathbf{Z}\} \\ &= c_\tau + \gamma_\tau x + \boldsymbol{\eta}_\tau'\mathbf{Z} + \sum_{k=1}^{p}\beta_{k,\tau}(c_k+\alpha_k x^* + \boldsymbol{\zeta}_k'\mathbf{Z}) \\ &= C_1 + \gamma_\tau x + \left(\sum_{k=1}^{p}\alpha_k\beta_{k,\tau}\right)x^* + \mathbf{C}_2'\mathbf{Z},\end{aligned}$$

where $\boldsymbol{\beta}_\tau = (\beta_{1,\tau},\cdots,\beta_{p,\tau})'$, and

$$\begin{aligned}C_1 &= c_\tau + \sum_{k=1}^{p} c_k\beta_{k,\tau}, \\ \mathbf{C}_2 &= \boldsymbol{\eta}_\tau + \sum_{k=1}^{p}\beta_{k,\tau}\boldsymbol{\zeta}_k.\end{aligned}$$

The first equality comes from the model assumption on $E[\mathbf{M}(\mathbf{x}^*)|\mathbf{Z}]$, and the second equality comes from the fact that conditioning on $\mathbf{Z}$, $E[\mathbf{M}(\mathbf{x}^*)|\mathbf{Z}]$ is fixed and thus the model for $Q_\tau\{Y(x,\mathbf{m})|\mathbf{Z}\}$ can be used by replacing $\mathbf{m}$ by $E[\mathbf{M}(\mathbf{x}^*)|\mathbf{Z}]$. This ends the proof.

3 Table A: Summary results of top five CpGs by Shen et al and Bind et al †.

Table A. Summary results of top five CpGs by Shen et al and Bind et al †.

$\tau = 0.2$	Shen et al	cg14541773	cg00063111	cg23576855	cg05227865	cg18625289
	Bind et al	cg12378760	cg09566177	cg00063111	cg23576855	cg18625289
$\tau = 0.3$	Shen et al	cg14541773	cg09566177	cg00063111	cg23576855	cg05575921
	Bind et al	cg09566177	cg00063111	cg23576855	cg05575921	cg18183624
$\tau = 0.4$	Shen et al	cg05575921	cg05227865	cg00376553	cg20652404	cg22590761
	Bind et al	cg05575921	cg05227865	cg00376553	cg23902550	cg03796381
$\tau = 0.5$	Shen et al	cg05575921	cg00028013	cg20652404	cg22590761	cg09090484
	Bind et al	cg05575921	cg23902550	cg03796381	cg20652404	cg07738730
$\tau = 0.6$	Shen et al	cg12876356	cg05575921	cg13468451	cg20652404	cg09090484
	Bind et al	cg03043406	cg12876356	cg18316974	cg05575921	cg23902550
$\tau = 0.8$	Shen et al	cg18968475	cg00713549	cg23902550	cg04581516	cg15106604
	Bind et al	cg03043406	cg05369582	cg00713549	cg23902550	cg04581516

† $\tau = 0.7$ is not presented as our method does not identify a statistically significant mediator at this quantile either.
